# Perceptibility and Acceptability of Tooth and Gingival Shade Modifications in Digital Smile Images: A Comparative Study Among Laypeople, General Dentists, and Specialists

**DOI:** 10.3390/dj13110534

**Published:** 2025-11-13

**Authors:** Nikola Petričević, Natalija Prica, Asja Čelebić, Sanja Peršić-Kiršić

**Affiliations:** 1 Department of Removable Prosthodontics, School of Dental Medicine, University of Zagreb, Gundulićeva 5, 10000 Zagreb, Croatia; celebic@sfzg.hr (A.Č.); persic@sfzg.hr (S.P.-K.); 2 Dental Clinic Zagreb, Perkovčeva 3, 10000 Zagreb, Croatia; nprica88@gmail.com

**Keywords:** perceptibility, acceptability, tooth, gingiva, shade, laypeople, general dentists, prosthodontist, periodontist, orthodontist

## Abstract

**Background:** This study aimed to evaluate the agreement among different evaluators in assessing smile esthetics from frontal-view photographs of the lower third of the face during smiling, and afterwards to determine thresholds of perceptibility and acceptability of tooth and gingival shade changes on a single modified digital photograph. **Methods:** Sixty photographs of the lower third of the face of individuals with pleasing smiles were obtained. Evaluator groups included laypeople, general dentists, and specialists in periodontology, orthodontics, and prosthodontics. Esthetic assessment was performed using seven items from the Orofacial Esthetic Scale (OES). One photograph was digitally manipulated by altering the shade of the first maxillary incisor and the gingiva of the right maxillary second incisor. Perceptibility thresholds and acceptability of these modifications were assessed by all evaluator groups. **Results:** Specialists in periodontology and prosthodontics, although rating 60 photographs as more esthetically pleasing, detected changes in tooth and gingival color earlier and judged such deviations as unacceptable sooner than general dentists and laypeople, particularly for shifts in lighter shades. Laypeople noticed color changes later but classified them as unacceptable almost immediately showing greater tolerance for lighter shades. **Conclusions:** The study shows that laypeople prioritize brighter tooth shades, whereas dental specialists value a more natural appearance. Specialists’ early detection of subtle shade changes and discerning judgments reflects their clinical training and awareness of the challenges in achieving perfect esthetics. In contrast, laypeople, seeking bright teeth influenced by social esthetic norms, noticed changes later but judged them as unacceptable more quickly.

## 1. Introduction

In contemporary dentistry, the esthetics of teeth and their surrounding structures are regarded as essential, contributing significantly to both health- and oral health-related quality of life [1,2,3]. Orofacial esthetics depends on multiple factors, including tooth color and brightness, tooth contour, position, shape, and size in addition to the texture and color of the gingiva, lips, buccal corridor visibility, and the morphology of the jaws [3,4,5,6,7,8]. However, the evaluation of orofacial esthetics is shaped by a variety of factors, such as sociodemographic variables, educational background, psychological and cultural factors, influences from media, etc. [9,10,11,12,13,14,15,16,17,18,19,20,21,22,23,24]. Although patient perception and attitudes play a significant role in determining what is esthetically pleasing, evaluations of dental professionals are also crucial for the comprehensive assessment of dental and facial esthetics. Successful dental/oral treatment largely depends on the clinician’s knowledge, experience, attitudes, and judgment [11,25,26,27]. Discrepancies between patient and dental professional perceptions can lead to unsatisfied patient, underlining the importance of assessing expectations before treatment [28,29,30,31,32,33,34,35]. Esthetic perception is a complex multifaceted process that can be evaluated using different instruments or observer-reported questionnaires. The Orofacial Esthetic Scale (OES), developed by Larsson et al., is a validated unidimensional instrument for assessing dental and orofacial esthetics [36,37,38]. It measures patients’ self-perception and has been psychometrically validated in multiple countries, including Croatia, demonstrating strong reliability and validity [39]. Other questionnaires related to orofacial esthetics have also been used, such as Questionnaire for Outcome Assessment of Smile Esthetic (OA-Smile), Dentofacial Appearance Perception Scale, Orofacial Appearance Perception Questionnaire, Psychosocial Impact of Dental Esthetics Questionnaire (PIDAQ), Dental Esthetic Screening Index, OHIP-ESTHET, etc. [40,41,42,43,44,45]. Other methods for evaluating esthetics employ dental photographs or video recordings, as well as digital image manipulations to simulate changes in tooth shape, alignment, dimensions, color, proportions, symmetry, and even treatment outcomes [8,14,16,46,47,48,49,50,51]. Changes in orofacial esthetic perception related to dental shade variations emphasize brightness as a primary factor in determining smile attractiveness and social appeal [52]. Computationally manipulated photographs with graduated brightness levels demonstrate that darker teeth are rated significantly less attractive than those with natural coloration [49,53,54]. Changes in tooth brightness strongly influence perceived social attractiveness, affecting social relationships, perceived happiness, and academic performance [49,53,55]. Laypeople generally prefer brighter teeth, whereas dentists favor more natural shades [3,8,56,57,58]. Moreover, differences in orofacial esthetic perception depending on changes in gingival brightness highlight the importance of pink esthetics alongside dental “white esthetics” in smile evaluation [59].

### Aim of the Study

This study aimed first to evaluate and compare agreement among laypeople, general dentists, and dental specialists in assessing smile esthetics from different frontal-view photographs of the lower third of the face of different individuals with a pleasing smile, and then to determine thresholds of perceptibility and acceptability of tooth and gingival shade changes among same groups using only one digitally manipulated photograph.

## 2. Materials and Methods

The study was conducted in accordance with the principles of the Declaration of Helsinki and was approved by the Institutional Ethics Committee of the authors’ affiliated institution. Written informed consent was obtained from all participants (volunteers and evaluators) prior to their inclusion in the study.

The first step was to obtain photographs of the lower third of the face from the volunteers (30 males and 30 females), 19–25 years old, with harmonious smiles, no orthodontic anomalies, and no restorations on visible teeth. Only small rotations of teeth were allowed. Subjects who had gingivitis, gum hyperplasia or recessions, as well as those who had any dental fillings on visible teeth, any crowns, or veneers, and those with orthodontic anomalies were not candidates to be photographed. The subjects were smiling, and the photographs of the lower third of the face were obtained to allow frontal teeth visibility. All photographed volunteers had been informed about the purpose and procedures of the study and provided their written informed consent for the use of photographs of their lower facial thirds in the research, with the assurance that their identities would remain confidential. Immediately before obtaining digital photographs, women removed all traces of lip makeup, and men shaved to ensure that evaluators could clearly see the teeth and lips, focusing on orofacial esthetics. Volunteers were photographed seated upright against a white wall, facing forward, with lips parted or smiling. Only the lower third of the face (with visible anterior teeth, gingiva, and lips) could be seen in their photographs. Before imaging, teeth were cleaned and polished.

A digital camera (Fujifilm X-Pro 3 camera, Tokyo, Japan) with a Fujinon XF50mmF2 R WR lens mounted on a tripod at a fixed distance (39 cm), with a gray reference card (WhiBal, Michael Tapes Design, Granite Falls, WA, USA) placed next to the face for color calibration was used [60]. Camera settings were ISO 100, 1/125 s shutter speed, f/2 aperture, 5500 K white balance, and 10 MP resolution. Images were acquired under controlled artificial lighting (neon tubes, light temperature 5080 K, 500 l×) verified with a Chroma-2 colorimeter (Lisun Electronics, Shanghai, China) [61]. Different groups—laypeople, general dentists, and specialists in Periodontology, Orthodontics, and Prosthodontics (Evaluators)—assessed esthetics of the obtained frontal-view photographs of the lower third of the face by using seven items of the Orofacial Esthetic Scale (OES). Assessments were made on a 5-point Likert scale (1 = completely dissatisfying, 5 = completely satisfying) [39]. Item no. 2 (esthetics of the facial profile of the lower third of the face) was excluded as only frontal-view photos were available. The assessed items in the photographs were (1) appearance of the lower third of the face, (2) mouth appearance (smile, lips, and visible teeth), (3) appearance of the rows of teeth, (4) shape of teeth, (5) color of the teeth, (6) gum’s appearance, and (7) the overall impression of the lower third of the face, mouth, and teeth.

The evaluators’ age ranged from 36 to 46 years. Before participation, all evaluators completed the Farnsworth–Munsell 100 Hue Test (X-Rite, Grand Rapids, MI, USA) to assess their color discrimination ability, and those with an error score above 20 were excluded from the study.

In the second step, 10 evaluators (2 from each group: laypeople, general dentists, periodontists, orthodontists, and prosthodontists), knowing the aim of this part of the study, scored the photographs from 1 to 5 (1-worst; 5-best) based on the suitability for manipulations. Those evaluators were excluded from assessing the photographs manipulated. In the next step, the chosen photograph was manipulated by changing shades of the first right maxillary incisor (brightness or darkness) and by changing shades of the second right maxillary incisor’s gingiva. The photograph which was original (not manipulated) was the reference photograph (P). Similar manipulations have already been described [62]. Additionally, the shade of tooth 11 of the chosen volunteer was measured in the mouth using a calibrated spectrophotometer (VITA Easyshade V, VITA Zahnfabrik, Bad Säckingen, Germany) on the middle third of the right maxillary central incisor, yielding CIE L*a*b* values: L = 84.8, a = −2.2, b = 14.6, i.e., A1 color.

**Figure 1:**
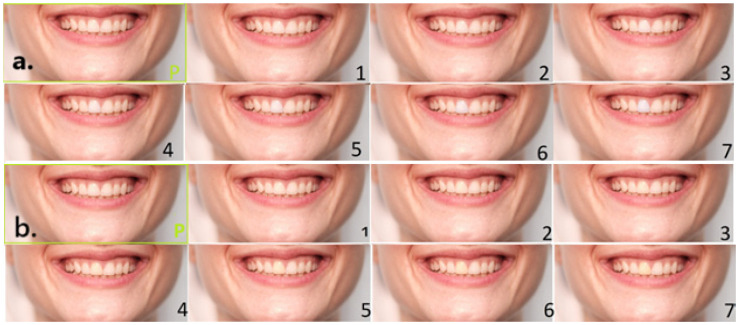
(**a**). Lighter shade variations in the right maxillary central incisor (tooth 11); (**b**). darker shade variations.

The shade manipulation of the first maxillary incisor was performed by changing white balance (WB), which was first increased in steps of 200 K from the original 5500 K to 6900 K to obtain darker shades (7 manipulated photographs, 5700 K, 5900 K, 6100 K, 6300 K, 6500 K, 6700 K, and finally 6900 K). After that, white balance was decreased, also in steps of 200 K from the original 5500 K to 4100 K to obtain lighter shades (brightness) (7 manipulated photographs). This yielded two sets of 8 photographs (Figure 1a,b). The original reference photograph was framed in green and marked “P”, while manipulations were numbered 1–7, in order of increasing or decreasing shade. Adobe Photoshop (v.21.0.0. Adobe Inc., San Jose, CA, USA) was used for manipulations. The incisor with a darker or lighter shade was cut out and inserted over the same tooth in the P and numbered according to a degree of shade increase or decrease.

**Figure 2:**
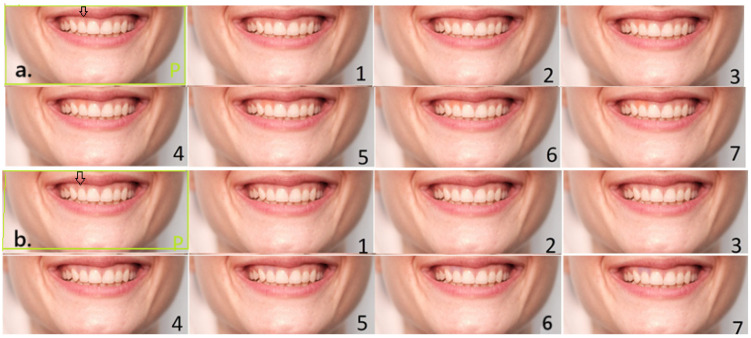
(**a**). Lighter shade variations in the gingiva of the right maxillary second incisor (tooth 12) with an arrow pointing to the place to be manipulated (on the original photograph); (**b**). darker shade variations.

Same procedure was obtained for the gingiva above the second maxillary incisor. It was also digitally manipulated in two directions, progressively lighter and progressively darker, each by 7 steps with 200 K increase or decrease in each step. The gingiva above tooth 12 was cut from the manipulated photographs (200 K difference from each other) and inserted into the original 5500 K image (Figure 2a,b). The original reference photograph was again framed in green and marked “P”, while manipulations were numbered 1–7. Each number represented an increase or a decrease of 200 K.

All sets of photographs (lighter and darker tooth shade, lighter and darker gingiva) contained 8 images each (original + 7 manipulations). For each set, evaluators had to indicate the first photograph when they noticed a change compared to the reference photograph. The second task was to indicate the photograph when the change became esthetically unacceptable. The same groups, laypeople, general dentists, and specialists in periodontology, orthodontics and prosthodontics participated in this second phase, assessing digitally manipulated images. The scores ranged from one to seven; lower values indicated that the evaluator either detected the change earlier or judged it as unacceptable at an earlier stage of manipulation, and vice versa. All judgements were performed on the same computer screen, not on paper photographs as in the first part of the study, because that enabled evaluators to zoom-in images to monitor more subtle details. The monitor was calibrated using Windows’ built-in display color calibration tool and online visual test patterns to standardize brightness, contrast, and color balance.

Sample size was calculated (alpha = 0.05, power = 80%) to define the number of participants (evaluators) based on a previous study [62]. The minimum number needed was 26 for each group, for a total of 130. However, the sample size of evaluators was intentionally increased to account for the possible exclusion of those with missing data.

### Statistical Analysis

Data were analyzed using SPSS software no. 21 (IBM Corp., Armonk, NY, USA). Descriptive statistics (means, standard deviations, and frequencies) were calculated for all variables. Gender distribution across evaluator groups was compared using the Chi-square test. Normality of distributions were assessed by the one-sample Kolmogorov–Smirnov test. Differences in mean values of the OES scores, and separately of tooth color ratings of 60 photographs, as well as perceptibility/acceptability thresholds for tooth and gingival shade changes in one manipulated photograph were tested using one-way ANOVA with Scheffé post hoc analysis. Statistical significance was set at *p* < 0.05.

## 3. Results

A total of 155 participants assessed the photographs divided into five groups; a total of 33 were laypeople, 30 were general dentists, 30 were specialists in periodontology, 30 were in orthodontics, and 32 were in prosthodontics. According to gender, there were 104 (67.1%) females and 51 (32.9%) males. No significant difference in gender distribution between the groups was found (Chi-square = 3.26; *p* = 0.53 not significant). All had similar ages.

Mean values and standard deviations of the assessed color of the visible teeth (and mean OES values) on the 60 photographs, evaluated by five groups (laypeople, general dentists, specialists in periodontology, orthodontics, and prosthodontics) are presented in Table 1.

No statistically significant differences were found among five groups assessing 60 different smile photographs, neither for a single question: assessment of anterior teeth color (F = 1.51; *p* = 0.20), nor for the mean value of the seven OES questions. However, laypeople gave the worst scores for both tooth color and the OES. Specialists in orthodontics and prosthodontics gave best scores for the tooth color on 60 photographs, as well as for overall assessment (OES).

Table 2 shows the mean values of the point when the participants (five groups: laypeople, general dentists, and specialists in periodontology, orthodontics, and prosthodontics) first noticed a change (darker color of the maxillary central incisor) on the manipulated photograph, and the point when they considered the change to be esthetically unacceptable. The significance of the differences between the groups is also shown (asterix). There was a significant difference between the five groups (F = 9.27, *p* < 0.01) in the initial detection of the darker incisor. All dentist groups noticed the darker incisor significantly earlier than the laypeople (Sheffe post hoc; *p* < 0.05). A significant difference was also observed for the assessment of when the darker shade became esthetically unacceptable (F = 5.34, *p* < 0.01), as the specialists in periodontology and prosthodontics assessed that the manipulation of increasing darker shades was no longer acceptable significantly earlier than the laypeople, general dentists, and specialists in orthodontics (Sheffe post hoc; *p* < 0.05).

Table 3 presents the mean values of the perceptibility threshold of a lighter shade change in the maxillary central incisor assessed by the five groups of participants, as well as mean values of the point when they considered the change esthetically unacceptable. There was a significant difference between the groups in the initial detection of the lighter incisor (F = 5.341, *p* < 0.01). Specialists in prosthodontics and periodontology were the first to notice lighter shade change compared to the other three groups (Sheffe post hoc; *p* < 0.05). Similarly, there was a significant difference in determining the point when the lighter shade was no longer considered acceptable (F = 4.70, *p* < 0.01). Specialists in prosthodontics assessed unacceptable lighter shade significantly earlier than laypeople and specialists in orthodontics (Sheffe post hoc; *p* < 0.05).

The results of the threshold when the darker gingiva was first observed, as well as of the point when the darker gingival shade of the second right maxillary incisor was no longer acceptable, assessed by the five groups (laypeople, general dentists, and specialists in periodontology, orthodontics, and prosthodontics) are presented in Table 4. A significant difference between the groups was found (F = 10.85; *p* < 0.01) for the initial detection of a darker gingiva. Laypeople noticed that the gingiva of the right second maxillary incisor was darker significantly later (more manipulations of the photograph) than other four groups of dentists (Scheffe post hoc; *p* < 0.05). A significant difference between the groups was also found for the mean values when darker gingiva was no longer acceptable (F = 4.62; *p* < 0.01); however, specialists in periodontology and prosthodontics considered darker shade unacceptable earlier than other groups (Scheffe post hoc, *p* < 0.05).

Mean values of the perceptibility threshold of the lighter gingiva of tooth 12 in the studied groups are presented in Table 5, as well as mean values when the lighter gingiva was no longer esthetically acceptable. There was a significant difference for the variable lighter gingival shade when it was first observed (F = 8.35; *p* < 0.01), as all three groups of specialists (in periodontology, orthodontics, and prosthodontics) noticed the change earlier than general dentists and laypeople (Scheffe post hoc, *p* < 0.05). Mean values of the variable “lighter shade is no longer acceptable” (F = 2.51) did not differ significantly among groups. However, specialists considered the lighter gingival shade change unacceptable a bit earlier than general dentists and laypeople.

## 4. Discussion

In the context of dental esthetics, dental practitioners are primarily guided by pragmatic principles aimed at enhancing overall oral and dental appearance, while the patients (laypersons) are mostly concerned by the appearance of teeth and their color [8,11]. Considering findings from various studies that highlight differences in esthetic perceptions between laypersons and dental professionals, an important question emerges: how can these divergent perspectives be reconciled to establish a compromise that adequately addresses the expectations of patients while remaining consistent with professional standards of dental practice?

To fulfill esthetic requirements, the clinician seeks to accommodate the patient’s expectations regarding esthetic outcomes in tooth brightness; however, discrepancies frequently arise between the dentist’s professional perception of esthetic harmony and the patient’s subjective preferences [33]. Furthermore, it should be acknowledged that a range of social, cultural, and psychological determinants, many of which remain insufficiently understood, also contribute to shaping the concept of the esthetic ideal within a given population [8,9,10,11,12,13,14,15,16,17,18,19,20,21,22,23,24,49,53,55].

Since evaluators—particularly laypeople—primarily observe others from a frontal perspective, where the teeth are most visible, only frontal-view photographs were included in the study. Therefore, profile photographs and item 2 of the Overall Esthetic Score (OES) questionnaire were excluded. The OES questionnaire serves as a validated instrument for assessing dental and orofacial esthetics, making it well-suited for evaluating patients’ self-perception [36,37,38]. However, in this study, the questions referred to photographs of other people, instead of on evaluation of their own self-perceived esthetic.

In the present study, when assessing esthetics of standardized photographs of different individuals with their own teeth without orthodontic anomalies and using the seven OES items for judging the photographs (Table 1), laypeople assigned the lowest scores for both tooth color and the Overall OES score, reflecting their prioritization of bright tooth color as one of the most important criteria when judging smile esthetics. In contrast, specialists in orthodontics and prosthodontics assigned the highest scores for tooth color across 60 standardized photographs, as well as for the overall OES assessment, reflecting a preference for a natural smile and a tendency to consider achievable outcomes in dental esthetic enhancement in terms of an optimally natural appearance. Laypeople, probably under the influence of media (TV, movies, magazines), anything that deviates, even slightly, from the established ideal, judge more strictly preferring brighter colors, while specialists in different dental fields, probably very small deviations from ideal, rate much better as they want to establish natural appearance [8,11,14,15,16,18,19,20,62,63,64,65,66,67,68,69].

Previous research in this field predominantly examined the perspectives of general population (laypeople) and general dental practitioners [8]. Fixed or removable denture fabrication is mostly performed by specialists in Prosthodontics, but it encompasses not only white but also pink esthetics and gingival treatments, and accounts for a substantial proportion of dental procedures from other dental specialists. The recent literature indicates that the central issue is not solely the existence of differences among the evaluator groups, but rather the magnitude of these differences and their potential clinical relevance. In this study, besides specialists in prosthodontics, specialists in periodontology and orthodontics were also included to examine potential differences in assessments, in comparison to general population and general dental practitioners. As previously discussed, these differences corresponded to variations in perception of anatomical features of anterior teeth and adjacent structures, with particular emphasis on the evaluation of tooth and gingival color and texture. For this assessment, dental photographs had to be standardized, which was performed for each photograph, as each one was obtained under same conditions by the same equipment, with white balance being calibrated at 5500 K [70]. It is well known that colors in different cameras or smartphones differ and cannot be completely accurate for the digital determination of tooth color [14,71,72]. However, if any difference in color (dE) existed in the assessed photographs, all of them presented the same shift.

To gain a more detailed understanding of the assessments made by each evaluator group for tooth color, the study included one photograph which was manipulated with a 200 K difference between each color adjustment. In contrast to previous studies, where the manipulation threshold was 400 K [14], this approach allowed more precise discrimination of the color change detection threshold and its variation across the five evaluator groups. However, some changes may be observed but still tolerated; therefore, the nonacceptance threshold was also observed.

When the right maxillary incisor was darkened, laypeople detected changes on average after the fourth manipulation (mean 4.21), corresponding to approximately 800 K of manipulation, whereas dental professionals perceived changes earlier, on average at manipulations between 400 and 600 K. Interestingly, periodontologists obtained the lowest mean value of 2.73, followed by prosthodontists with a mean value of 2.81. It is also interesting that the lowest observed value reported in all dental professional groups was 1, corresponding to only 200 K change, while the lowest observed value in the laypeople group was 2 (400 K). To control individual differences in possible perceptual thresholds for tooth color, the FM Hue test excluded all participants with error scores above the first quartile. Therefore, lower perception threshold of the darker tooth 11 (200 K: 400 K) of dental professionals compared to laypeople is probably not due to their inherited discrimination ability, but to their focus, knowledge, and experience. The highest discriminative threshold in laypeople was 7 (1400 K), while in dental professionals, the highest threshold varied between 4 and 6 (800–1200 K). Indeed, the ability to detect a change in tooth color toward a darker shade and the point at which this change is no longer considered acceptable is small and is lowest among laypeople (Table 2).

Conversely, for changes toward a lighter shade, all groups detected differences sooner: laypeople had a mean detection value of 2.83 (corresponding to nearly 600 K of color manipulation), while professionals detected changes earlier at mean values ranging from 2.0 to 2.37, corresponding to 400–500 K toward lighter shades. Minimum observed change threshold was 1 (200 K) for all groups. The minimum value at which the change was no longer acceptable was also 1, as was the perceptive threshold. Mean value of non-acceptance was a bit higher in the laypeople group compared to professionals, particularly specialists in prosthodontics and periodontology who judged even a bit smaller lighter shade changes to be unacceptable.

Generally, professionals are more tolerable towards darker than lighter tooth shade changes as they judge lighter shades as not natural. Laypeople, under the influence of the media, show much tolerance towards lighter shades. The widespread interest in tooth whitening within the general population supports findings that this group prioritizes lighter color as the primary esthetic criterion. On the contrary, only minimum difference existed in laypeople between the thresholds of perceptibility and non-acceptability of tooth shade manipulation towards darker shades. Similar findings were reported in other studies (preclinical and clinical students were included), confirming importance of clinical education to notice changes earlier [14,62].

For darker or lighter shade manipulations of the gingiva above the second right maxillary incisor, laypeople detected changes on average at approximately 800 K, whereas professionals detected between 400 and 600 K. When the gingiva was darkened, laypeople detected changes only after the fourth manipulation (value of 4.15), corresponding to approximately more than 800 K of manipulation, whereas dental professionals perceived changes earlier at manipulations between 400 and 600 K. As expected, periodontologists were the first to recognize the presence of darker gingival coloration, with a mean value of 2.60. Prosthodontists also demonstrated a notable perceptibility of gingival color change, with a mean value of 2.88. This can be attributed to the professional training in both specializations and the challenges posed by endodontically treated roots and the subsequent need to mask the grayish effect on both gingiva and crowns in prosthodontic restorations. Comparable results were observed across all examiner groups when assessing changes toward lighter gingival shades.

Interestingly, specialists in prosthodontic provided almost the highest esthetic ratings of the lower third of the face and visible teeth in the first part of the study (Table 1), while in the second part (Table 2, Table 3, Table 4 and Table 5), together with periodontists, they were the first to detect color changes in manipulated photographs and the first to judge changes as esthetically unacceptable, especially when the lighter shade was manipulated. This may be explained by their clinical focus on creating restorations that mimic natural appearance with minor esthetic irregularities, while they considered altered tooth or gingival color in the manipulated images, especially toward lighter shades, no longer natural and therefore rated them as less acceptable. Another explanation may be that the specialists’ extensive training makes them both more sensitive to changes in tooth color and more aware of the limitations of dental procedures in achieving a natural appearance. Consequently, they are more flexible in accepting minor irregularities and imperfections in the smile preferring natural appearance than ideal.

In contrast, laypeople gave the lowest esthetic ratings in the first part of the study (Table 1) but detected changes in tooth or gingival color significantly later than dental professionals, regardless of whether the shade shifted toward darker or lighter tones. However, once they noticed a difference, they almost immediately judged that as unacceptable (Table 2, Table 3, Table 4 and Table 5). Dentists, on the other hand, perceived the changes much earlier but showed a wider gap between the point of detection and the point at which they classified the change as unacceptable. This difference can be attributed to professional training during their study [8,14,15,16,21,25,30,33,46,48,49,57,59] and experience in clinical practice [11,57], which enable dentists to detect subtle shade variations earlier. Laypeople, lacking such training, perceive manipulated changes later, but they tend to classify detected differences as unacceptable without tolerance because media and social standards strongly promote bright, regular teeth as ideal, which is also confirmed by the results of the first part of the study. Additionally, general dentists and orthodontists showed intermediate responses, detecting shade changes earlier than laypeople but later than prosthodontists and periodontists (Table 2, Table 3, Table 4 and Table 5). Their broader tolerance between the point of detection and the point at which the change was determined unacceptable may reflect their focus on function and alignment rather than subtle color discrepancies. Overall, professional education and clinical experience clearly enhanced the ability to detect shade variations earlier, while laypeople’s judgments were strongly influenced by social esthetic norms. Moreover, the social media landscape has become a powerful determinant of patients’ esthetic aspirations, often leading individuals, especially younger populations, to request treatments primarily aimed at cosmetic rather than functional or health-related improvement. These trends may also alter the dynamics of the dentist–patient relationship, as clinicians are increasingly confronted with patients whose requests are shaped by online imagery rather than by realistic clinical outcomes. Consequently, the perception of what is considered “esthetically acceptable” can be distorted by digital representations rather than by biological or functional harmony [68,73].

However, limitations of the study must be acknowledged. In the first part of the study, although the photographs were standardized, they could still contain errors due to the characteristics of the camera. Nevertheless, these characteristics were consistent across all photographs because of the standardization process. Another limitation is that the calibration of the computer monitor may vary according to lighting in their working area. Also, the monitor’s color gradually changes over time, even though it is not evident for most humans. Ambient lighting affects how we perceive colors, so any changes to in the environment require screen recalibration. Although there are several ways of standardizing computer screens to minimize possible errors, we decided to use only one computer screen for all evaluators. Only Windows’ built-in software, i.e., the color calibration tool, was utilized. Future studies may perform hardware calibration for improved accuracy as well.

The evaluations were conducted simultaneously (11:00–12:00) under identical lighting conditions. However, factors such as fatigue, motivation, or other psychological distractions may have influenced the perceptibility of color changes and the threshold at which such changes were deemed unacceptable. Future studies should randomize image sets and provide breaks between viewing sessions to reduce fatigue or sequence bias.

Another limitation is the use of the OES scale, which was originally designed for self-assessment of personal appearance. In this study, it was applied to evaluate photographs of other individuals. This methodological adaptation could affect the scale’s validity and should be further studied.

## 5. Conclusions

The present study shows that laypeople prioritize brighter tooth color when judging smile esthetics, whereas dental specialists value a more natural appearance and rate minor deviations from the ideal more favorably. This likely also reflects the specialists’ awareness on the difficulty of achieving natural appearance, while the general population tends to seek idealized perfection. The study also shows that dental professionals detect subtle gingival and tooth shade changes earlier and judge them with greater nuance due to professional training and clinical experience, while laypeople notice changes later but judge them as unacceptable more quickly, reflecting social esthetic ideals rather than natural appearance considerations.

## Figures and Tables

**Table 1:** Mean values and standard deviations of the assessed color of the visible teeth on standardized photographs, together with mean OES values, among five groups.

Variable		N	Mean	Std. Deviation
Teeth color	Laypeople	33	3.51	0.48
	General dentists	30	3.63	0.36
	Specialists in Periodontology	30	3.52	0.65
	Specialists in Orthodontics	30	3.75	0.54
	Specialists in Prosthodontics	32	3.72	0.43
Mean value of the seven OES items	Laypeople	33	3.52	0.46
	General dentists	30	3.59	0.33
	Specialists in Periodontology	30	3.52	0.42
	Specialists in Orthodontics	30	3.66	0.51
	Specialists in Prosthodontics	32	3.63	0.51

N = number of participants.

**Table 2:** Mean values of the point when darker incisor shade (tooth 11) was first noticed (perceptibility threshold) and the point when the darker change was considered no more esthetically acceptable, together with minimum and maximum values and significance of the differences between laypeople, general dentists, and specialists in periodontology, orthodontics, and prosthodontics.

		Mean	SD	Min.	Max.	F	*p*	*LP GD S-Perio SO S-Prosth*				
*Darker tooth shade first observed*	Laypeople	4.21	1.56	2.00	7.00	9.27	<0.01		*	*	*	*
	General dentists	2.90	1.03	1.00	6.00			*				
	Specialists in Periodontology	2.73	0.83	1.00	4.00			*				
	Specialists in Orthodontics	3.07	0.94	1.00	5.00			*				
	Specialists in Prosthodontics	2.81	1.15	1.00	6.00			*				
*Darker tooth shade no longer esthetically acceptable*	Laypeople	4.48	1.39	2.00	7.00	5.34	<0.01					
	General dentists	4.00	1.31	2.00	7.00							
	Specialists in Periodontology	3.43	0.90	2.00	5.00			*	*		*	
	Specialists in Orthodontics	4.07	1.14	2.00	7.00							
	Specialists in Prosthodontics	3.25	1.30	1.00	5.00			*	*		*	

SD = Standard deviation; Min. = minimum; Max. = maximum; F = F value; *p* = level of significance; *LP* = Laypeople; *GD* = General dentists; *S-Perio* = Specialists in Periodontology; *SO* = Specialists in Orthodontics; *S-Prosth* = Specialists in Prosthodontics; * = significant difference.

**Table 3:** Mean values of the point when lighter incisor shade (tooth 11) was first noticed and the point from which the lighter change was considered esthetically unacceptable, together with minimum and maximum values and significance of the differences between laypeople, general dentists, and specialists in periodontology, orthodontics, and prosthodontics.

		Mean	SD	Min.	Max.	F	*p*	*LP GD S-Perio SO S-Prosth*				
*Lighter tooth shade first observed*	Laypeople	2.82	1.07	1.00	5.00	3.67	<0.01			*		*
	General dentists	2.37	0.96	1.00	4.00							
	Specialists in Periodontology	2.00	0.95	1.00	4.00			*				
	Specialists in Orthodontics	2.37	0.93	1.00	4.00							
	Specialists in Prosthodontics	2.03	0.97	1.00	5.00			*				
*Lighter tooth shade no longer esthetically acceptable*	Laypeople	3.33	1.41	1.00	7.00	4.70	<0.01					*
	General dentists	3.00	1.14	1.00	6.00							
	Specialists in Periodontology	2.80	1.06	1.00	5.00			*				
	Specialists in Orthodontics	3.17	1.05	2.00	6.00							*
	Specialists in Prosthodontics	2.22	0.87	1.00	4.00			*			*	

SD = Standard deviation; Min. = minimum; Max. = maximum; F = F value; *p* = level of significance; *LP* = Laypeople; *GD* = General dentists; *S-Perio* = Specialists in Periodontology; *SO* = Specialists in Orthodontics; *S-Prosth* = Specialists in Prosthodontics; * = significant difference.

**Table 4:** Mean values of the point when darker incisor gingival shade (tooth 12) was first noticed (perceptibility threshold) and the point when the darker change was considered no more esthetically acceptable, together with minimum and maximum values and significance of the differences between laypeople, general dentists, and specialists in periodontology, orthodontics, and prosthodontics.

		Mean	SD	Min.	Max.	F	*p*	*LP GD S-Perio SO S-Prosth*				
*Darker gingival shade first observed*	Laypeople	4.15	1.37	1.00	7.00	10.85	<0.01		*	*	*	*
	General dentists	3.03	0.93	2.00	5.00			*				
	Specialists in Periodontology	2.60	1.00	1.00	5.00			*				
	Specialists in Orthodontics	3.30	0.79	2.00	5.00			*				
	Specialists in Prosthodontics	2.88	0.87	1.00	5.00			*				
*Darker gingival shade no longer esthetically acceptable*	Laypeople	4.33	1.27	2.00	7.00	4.62	<0.01					*
	General dentists	4.00	1.26	1.00	6.00							
	Specialists in Periodontology	3.47	1.14	1.00	6.00							
	Specialists in Orthodontics	4.00	0.91	2.00	6.00							
	Specialists in Prosthodontics	3.22	1.29	1.00	5.00			*				

SD = Standard deviation; Min. = minimum; Max. = maximum; F = F value; *p* = level of significance; *LP* = Laypeople; *GD* = General dentists; *S-Perio* = Specialists in Periodontology; *SO* = Specialists in Orthodontics; *S-Prosth* = Specialists in Prosthodontics; * = significant difference.

**Table 5:** Mean values of the point when lighter gingival shade (tooth 12) was first noticed and the point from which the lighter change was considered esthetically unacceptable, together with minimum and maximum values and significance of the differences between laypeople, general dentists, and specialists in periodontology, orthodontics, and prosthodontics.

		Mean	SD	Min.	Max.	F	*p*	*LP GD S-Perio SO S-Prosth*				
*Lighter gingival shade first observed*	Laypeople	4.36	1.48	2.00	7.00	8.35	<0.01			*	*	*
	General dentists	3.57	1.10	2.00	6.00							
	Specialists in Periodontology	2.87	1.17	1.00	5.00			*				
	Specialists in Orthodontics	3.37	1.00	2.00	5.00			*				
	Specialists in Prosthodontics	3.00	0.95	1.00	5.00			*				
*Lighter gingival shade no longer esthetically acceptable*	Laypeople	4.64	1.52	2.00	7.00	2.51	<0.05					
	General dentists	4.43	1.19	1.00	7.00							
	Specialists in Periodontology	3.93	1.68	1.00	7.00							
	Specialists in Orthodontics	4.00	1.20	2.00	7.00							
	Specialists in Prosthodontics	3.66	1.41	1.00	7.00							

SD = Standard deviation; Min. = minimum; Max. = maximum; F = F value; *p* = level of significance; *LP* = Laypeople; *GD* = General dentists; *S-Perio* = Specialists in Periodontology; *SO* = Specialists in Orthodontics; *S-Prosth* = Specialists in Prosthodontics; * = significant difference.

## Data Availability

The original contributions presented in this study are included in the article. Further inquiries can be directed to the corresponding author.

## Abbreviations

The following abbreviations are used in this manuscript:

OES	Orofacial Esthetic Scale
OA-Smile	Outcome Assessment of Smile Esthetic
PIDAQ	Psychosocial Impact of Dental Esthetics Questionnaire
OHIP	Oral Health Impact Profile
